# Inflammatory and resolution stages of hepatic injury: imaging with USPIO-enhanced MRI in mice

**DOI:** 10.1186/s41747-026-00701-6

**Published:** 2026-04-10

**Authors:** Joao Piraquive Agudelo, Sabrina Doblas, Katell Peoc’h, Bich-Thuy Doan, Philippe Garteiser, Bernard E. Van Beers

**Affiliations:** 1 Laboratory of Imaging Biomarkers, Center for Research on Inflammation, Inserm U1149, Université Paris Cité, Paris, France; 2 Department of Biochemistry and Nutrition, Beaujon University Hospital Paris Nord, AP-HP, France and UMR 1149 Inserm, Paris, France; 3 Unité de Technologies Chimiques et Biologiques pour la Santé, Université Paris Cité, UMR 8258 CNRS, Paris, France; 4 Department of Radiology, Beaujon University Hospital Paris Nord, AP-HP, Clichy, France

**Keywords:** Inflammation, Liver, Macrophages, Magnetic iron oxide nanoparticles, Magnetic resonance imaging

## Abstract

**Objective:**

Liver injury includes inflammation and resolution stages with different macrophage populations. Hepatic macrophages can be imaged with ultrasmall paramagnetic iron oxide (USPIO) nanoparticles-enhanced magnetic resonance imaging (MRI). We aimed to assess if inflammation and resolution stages of liver injury could be differentiated with USPIO-enhanced MRI in mice.

**Materials and methods:**

Three groups of C57BL/6JRj mice (control, inflammation, resolution; *n* = 10 for each group) were imaged. Liver fibrosis was induced by intraperitoneal carbon tetrachloride injections for 6 weeks. Multigradient-echo MRI was performed before, 24, and 48 h after intravenous injection of fluorescent USPIO. Contrast uptake was quantified with $\triangle {R}_{2}^{* }$ measurements. Macrophages were immunostained with F4/80, and USPIO fluorescence was localized and quantified with confocal microscopy. Liver iron content was measured with inductively coupled mass spectrometry (ICP-MS). $\triangle {R}_{2}^{* }$ was assessed with Mann–Whitney tests. Kruskal–Wallis and Dunn tests compared fibrosis, fluorescence, and ICP-MS iron concentration. Spearman tests were used for correlation analysis.

**Results:**

$\triangle {R}_{2}^{* }$ was significantly higher in the inflammation group (158 ± 85%) compared to the control (58 ± 36%, *p* = 0.020) and resolution (71 ± 36%, *p* = 0.048) groups. Confocal microscopy showed a high macrophage number and USPIO uptake in the inflammation group. $\triangle {R}_{2}^{* }$ correlated with macrophages number (*r* = 0.67, *p* = 0.0001), USPIO fluorescence intensity (*r* = 0.58, *p* = 0.0011), and iron concentration at ICP-MS (*r* = 0.39, *p* = 0.028).

**Conclusion:**

Our results suggest that the inflammatory and resolution stages of hepatic injury can be assessed with USPIO-enhanced MRI.

**Relevance statement:**

Our study demonstrates that USPIO-enhanced MRI can be used to monitor the inflammatory and resolution phases of hepatic injury in mice. If future studies confirm these findings, this imaging method might be valuable for tracking hepatic inflammation dynamics.

**Key Points:**

- Liver $\triangle {R}_{2}^{* }$ was highest during the inflammatory stage and partially reversed during the resolution stage, reflecting macrophage dynamics.
- Immunofluorescence showed increased macrophage number and uptake during inflammation, decreasing during resolution.
- Iron concentration significantly correlated with $\triangle {R}_{2}^{* }$, macrophage number, and total fluorescence intensity.

**Graphical Abstract:**

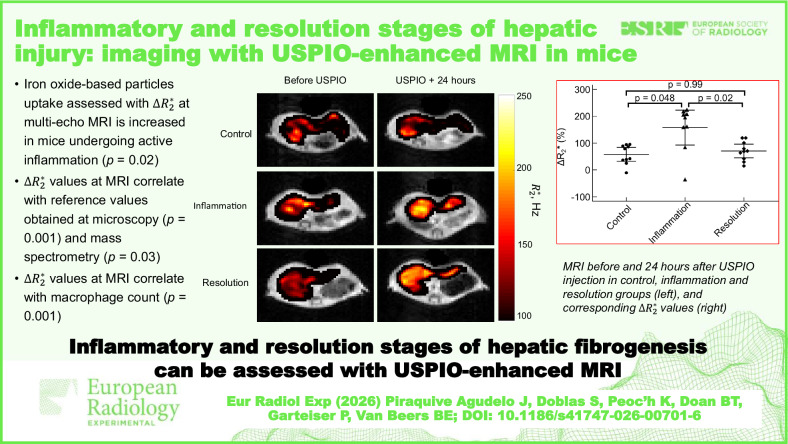

## Background

Hepatic injury is a dynamic process in which hepatic macrophages play a key role [1]. Hepatic macrophages are heterogeneous cell populations consisting of liver-resident Kupffer cells and monocyte-derived macrophages recruited from the circulation to the liver. During hepatic injury, monocytes are massively attracted to the liver, and monocyte-derived macrophages represent a large macrophage population with different phenotypes. In murine models, Ly6C^hi^ macrophages display a proinflammatory phenotype, while Ly6C^low^ macrophages show pro-resolution properties. In the first step, Ly6C^hi^ macrophages predominate. They exacerbate inflammation and promote the activation of hepatic stellate cells to become myofibroblasts, the main source of collagen. In a second step, recently infiltrated Ly6C^hi^ macrophages can transform into Ly6C^low^ macrophages, secreting matrix-degrading metalloproteinases and showing restorative and anti-inflammatory properties [2–4].

Hepatic macrophages thus have a two-faced phenotype, encouraging inflammation and fibrosis yet also supporting restoration and inflammation resolution [4]. Differentiating these two stages of liver injury may have prognostic and therapeutic consequences. Indeed, treatments aiming at inhibiting macrophage recruitment and activation [5] or stimulating macrophage switch to restorative macrophages [4, 6] have been proposed.

At ultrasmall paramagnetic iron oxide particle (USPIO)-enhanced MRI, liver signal intensity enhancement and *R*_2_* are altered in diffuse liver diseases when compared to those observed in the normal liver. These changes have been reported to be related to changes in macrophage population size and endocytic capacity [7–12]. *In vitro* studies suggest higher uptake of USPIO in proinflammatory than in pro-resolution macrophages [13, 14].

The aim of our study was to assess in a model of toxic liver injury induced by carbon tetrachloride (CCl_4_) in mice whether the inflammatory and resolution stages of hepatic injury could be differentiated with USPIO-enhanced MRI, based on changes in the number and endocytic capacity of hepatic macrophages.

## Materials and methods

### Animal model

The research protocol was approved by the Paris-Nord animal experimentation ethics committee (APAFIS#14088-2018031423143505v4), and the experiments were performed following the Animal Research Reporting of *In Vivo* Experiments‒ARRIVE guidelines [15]. Three groups (control, inflammatory, and resolution groups, *n* = 10 per group) of male C57BL/6JRj mice (Janvier Labs) were used for the study. Males were used as it has been reported that they are more susceptible to developing liver fibrosis than females [16, 17]. Liver inflammation and fibrosis were induced with intraperitoneal injections of CCl_4_ (0.5 mL/kg) in olive oil at a dilution of 1:10. Two CCl_4_ injections per week were performed for 6 weeks. The mice of the control group were age-matched and did not receive any CCl_4_ or oil injection. MRI was started by performing an unenhanced liver MRI followed by USPIO intravenous injection 4 h after the last CCl_4_ injection in the inflammatory group and 24 h after the last injection in the resolution group. USPIO-enhanced MRI was performed 24 h after USPIO injection in all mice. This means that MRI in the resolution group was performed 24 h and 48 h after the last CCl_4_ injection. In this mouse model, these time points correspond to previously reported peak concentrations of proinflammatory Ly6C^hi^ and pro-resolution Ly6C^low^ macrophages, respectively [17].

### MRI

The mice were anesthetized with isoflurane (1‒2% in 50%–50% mixture of oxygen and air) for imaging. MRI was performed on a 7-T Bruker system (BioSpec 70/30 USR, Bruker) with a 40-mm volume radiofrequency coil. Transverse sections of the liver were imaged with a two-dimensional multi-echo gradient echo sequence and the following parameters: echo time/repetition time 1.2/800 ms, 16 echoes, interecho delay 0.8 ms, half-Gaussian pulse of 0.8 ms, flip angle 19°, field of view 50 × 50 mm^2^, matrix size 64 × 128, number of slices 5, slice thickness 1 mm, no gap between slices, number of averages 2, bandwidth 100 kHz, and respiratory triggering. Another sequence was also acquired at a higher resolution for the purpose of providing anatomical reference images (ultrashort echo time used with the “half-pulse excitation plus FID” acquisition mode), echo time/repetition time 59 µs/10 ms, Half-Gaussian pulse of 0.75 ms, flip angle 15°, field of view 40 × 40 mm², 402 radial profiles, matrix size 128 × 128, number of slices 1, slice thickness 1.5 mm, number of averages 2, bandwidth 100 kHz).

Bimodal USPIOs (P01240, CheMatech) with a hydrodynamic diameter of 25‒30 nm and T_2_ relaxivity of 94 mM^-1^ s^-1^ (at 7 T and 37 °C in 4% human serum albumin) were used as a contrast agent. The bimodal USPIOs contain > 5,000 iron atoms and 7 rhodamine-B molecules per particle. USPIO-enhanced MRI was performed with 200 µmol of iron per kilogram of body weight. This dose was chosen as the smallest dose of the interval recommended by the manufacturer (200‒1,000 µmol/kg), itself based on the 1,000 µmol/kg dose previously used with the similar contrast agent P904 [18, 19].

### Image analysis

The region of interest (ROI) was placed in a homogeneous area of the right liver lobe. The ROI were drawn manually, avoiding blood vessels and organ edges by a physicist with 2-years of experience in abdominal MRI. The ROI size was 27 ± 6 mm^2^ and the number of pixels 109 ± 14 (mean ± standard deviation).

${T}_{2}^{* }$ quantification was performed by Levenberg-Marquardt minimization of the measured signal against the expression $\left({S}_{0}\cdot {e}^{-{TE}/{T}_{2}^{* }}\right)$ (Eq. 1), with ${S}_{0}$ and ${T}_{2}^{* }$ as free parameters. Minimization was performed with the lmfit C + + library (version 8.3) under an in-house graphical user interface coded in Matlab (The Mathworks). Values were calculated for each pixel in the ROIs and averaged. The relaxation rates (${R}_{2}^{* }=1/{T}_{2}^{* }$) (Hz) (Eq. 2) were calculated and used for further analysis. The delta ${R}_{2}^{* }$ ($\Delta {R}_{2}^{* }$) before and after USPIO injection were evaluated as (Eq. 3):

3 $$\Delta {R}_{2}^{* } = \frac{{R}_{2}^{* \,{post}}-{R}_{2}^{* \,{pre}}}{{R}_{2}^{* \,{pre}}}\times 100$$

### Histological analysis

The mice were sacrificed immediately after contrast-enhanced MRI. The right hepatic lobe was dissected and fixed in 10% buffered neutral formalin, embedded in paraffin, and sectioned at 5 µm. We used the right liver lobe for histological analysis, as we also used it for MRI assessment (see above). The liver slices were stained with picrosirius red and scanned with 20-fold magnification. The image J software (1.51J8; National Institutes of Health) was used for collagen percentage quantification. Images were split into red, green, and blue (RGB) channels. From the green channel, the red-stained collagen was isolated via thresholding. The threshold value was kept the same for all the study groups.

### Immunofluorescence

Mouse liver sections were deparaffinized by immersing the slides in two consecutive xylene baths for 5 min each. The sections were rehydrated through a graded ethanol series. Slides were rinsed in distilled water for 5 min. For permeabilization, sections were washed in 1× phosphate-buffered saline (PBS) containing 0.3% Triton X-100 for 5 min. This was followed by two washes in 1× PBS with 3% bovine serum albumin for 5 min each. Slides were placed in a humid chamber and blocked with 1× PBS containing 3% bovine serum albumin and 3% goat serum for 1 h. Sections were stained for the murine macrophage marker F4/80 with rat anti-mouse F4/80 antibody at a dilution of 1:20 (Invitrogen). Alexa Fluor 488 conjugated goat anti-rat IgG dilution 1:50 (Invitrogen) was used as a secondary antibody. Hoechst dye (Invitrogen; dilution 1:1000) was added for labeling nuclei. A Leica TCS SP8 confocal microscope (Leica Microsystems) was used for image acquisition, and ImageJ was used for image quantitation [20].

Rhodamine B of the fluorescent USPIOs was excited with a 561 nm diode-pumped solid-state laser, and the emission was filtered with a 568‒618 nm band-pass filter. The macrophage membranes labeled with F4/80 were excited with a 488 nm argon laser, and the emission was filtered with a 501‒555 nm band pass filter. The nuclei were excited with a 405 nm argon laser, and emission was captured with a 412‒447 nm band-pass filter. Six RGB images per mouse liver section were acquired with an oil immersion objective. Each image was acquired with 40-fold magnification, 1,952 × 1,952 matrix, 700-Hz scan speed, bidirectional mode for acquisition time reduction, 4-line averages, and pixel size 71 × 71 nm^2^.

For immunofluorescence analysis, six RGB images were created per mouse, with the red channel showing the USPIOs, the green channel showing the macrophages, and the blue channel showing the nuclei.

Macrophages having internalized USPIO were detected by green and red fluorescence co-localization. The internalized USPIO fluorescence intensity was determined after drawing ROIs around the red areas within the macrophages over the analyzed tissue region, and normalized relative to the physical surface of the analyzed tissue region. The total number of phagocytic macrophages was also derived at this step, and likewise normalized by surface. Finally, the mean fluorescence intensity was also evaluated in terms of fluorescence intensity per macrophage to evaluate the specific uptake of fluorescence by the macrophage population.

### Inductively coupled plasma mass spectrometry

Liver samples were desiccated for 15 h at 120 °C. Dried samples were weighed and mineralized with nitric acid solution (Optima Grade, Thermo Fisher Scientific) for 1 h at 110 °C in a microwave (Mars 6, CEM Corporation). The mineralized product was stored at 4 °C until assayed. Trace elements were measured by inductively coupled mass spectrometry (ICP-MS), on an X-Series II from Thermo Fisher Scientific equipped with collision cell technology. The source of plasma was argon (Messer) with a high degree of purity (99.99%). The collision/reaction cell was pressurized with a mixture of helium (93%) and hydrogen (7%) (Messer). Ultrapure water was obtained from a Direct-Q 3 water station (Millipore). Nitric acid solution was supra-pure, 69% (Optima Grade, Thermo Fisher Scientific). The internal standard used was rhodium (Thermo Fisher Scientific). Calibration ranges were performed with a multi-element calibrator solution (Plasmacal, SCP Science). Calibration and verification of instrument performance were realized with multi-element solutions, respectively, tune F and tune A (Thermo Fisher Scientific). Certified reference materials were obtained from NCS (bovine liver ZC71001) (NCS, Beijing, China).

### Statistical analysis

Sample size estimation was based on power calculation according to previous results of USPIO $\Delta {R}_{2}^{* }$ in patients with chronic liver disease [12]. In that previous study, $\Delta {R}_{2}^{* }$ difference between normal and diseased livers of 35 Hz with a standard deviation of 20 Hz was observed. With these parameters, group sizes of at least *n* = 7 mice were needed to provide 80% power with α = 0.05. Because of concerns regarding potential mouse mortality during the CCl_4_ induction period and the MRI procedures, we included ten mice in each group [21].

Data were expressed as mean ± standard deviation. Delta ${R}_{2}^{* }$ before and after intravenous injection of USPIO were assessed in the three groups (control, inflammation, and resolution groups) with Mann–Whitney tests. For each measured variable ($\Delta {R}_{2}^{* }$, fibrosis percentage, fluorescence, and ICP-MS iron concentration), Kruskal–Wallis and Dunn *post-hoc* tests were used to measure differences between the three groups. Correlation analysis was performed with Spearman tests. Differences between groups were considered significant at a *p* value threshold of 0.05. Data were analyzed with GraphPad Prism 7 (GraphPad Software, Inc.).

## Results

### MRI

**Fig. 1:**
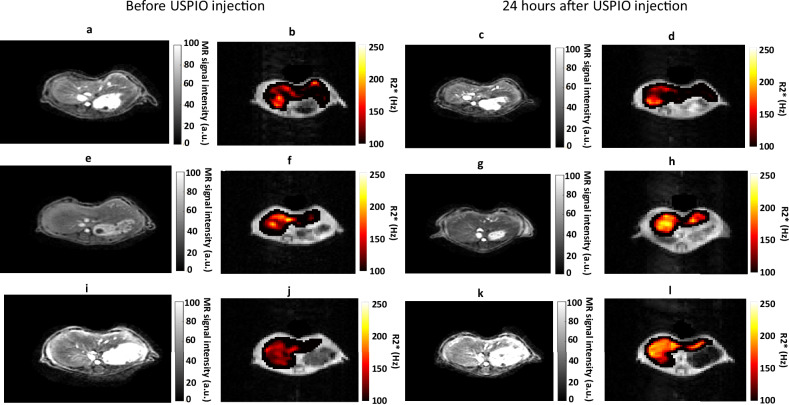
Liver ultrashort echo time (echo time/repetition time 59 µs/10 ms) anatomical reference images (**a**, **c**, **e**, **g**, **i**, **k**) and ${R}_{2}^{* }$ maps overlayed on the first echo image of the multi-echo gradient echo sequence (**b**, **d**, **f**, **h**, **j**, **l**) before and 24 h after injection of USPIO in control mouse (**a**–**d**), mouse during inflammatory stage (**e**–**h**) and mouse during resolution stage (**i**– **l**). A higher liver signal drop 24 h after USPIO injection relative to preinjection is observed in the inflammation stage than in the resolution stage and in the control mouse. Concomitant ${R}_{2}^{* }$ increase is higher in the mouse with inflammation than in the two other mice. USPIO, Ultrasmall paramagnetic iron oxide

**Fig. 2:**
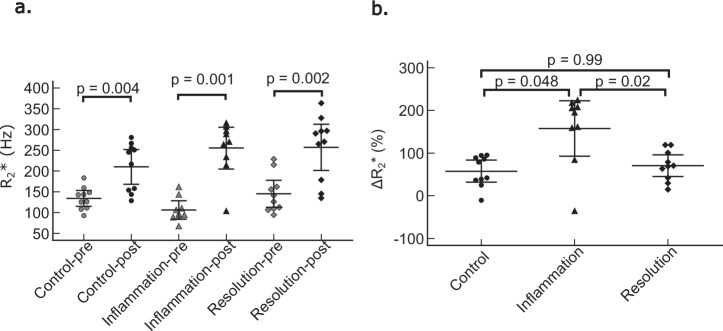
Hepatic ${R}_{2}^{* }$ relaxation rates before and after intravenous injection of USPIO (**a**) and $\triangle {R}_{2}^{* }$ measurements (**b**) in control mice and in mice during inflammation and resolution. Significant increases in ${R}_{2}^{* }$ are observed after USPIO injection in (**a**). $\triangle {R}_{2}^{* }$ is significantly higher in the inflammation group than in the resolution and control groups (**b**). USPIO, Ultrasmall paramagnetic iron oxide

Twenty-four hours after administration of USPIO, liver ${R}_{2}^{* }$ increased significantly in all mice (controls: 134 ± 27 Hz before USPIO injection *versus* 210 ± 58 Hz after injection, *p* = 0.004; inflammation group: 107 ± 29 Hz *versus* 255 ± 66 Hz, *p* = 0.0005; resolution group: 145 ± 14 Hz *versus* 201 ± 257 Hz, *p* = 0.002) (Figs. 1 and 2). Delta ${{{R}}}_{2}^{* }$ differed significantly between the 3 mouse groups (*p* = 0.011). Delta ${R}_{2}^{*}$ was significantly higher in the inflammation group compared to the control group (158 ± 85% *versus* 58 ± 36%, Dunn *post-hoc* test, *p* = 0.020) and the resolution group (71 ± 36%, *p* = 0.048). No significant difference was found between the control and the resolution groups (*p* = 0.423) (Fig. 2).

### Quantitative histology of liver fibrosis

**Fig. 3:**
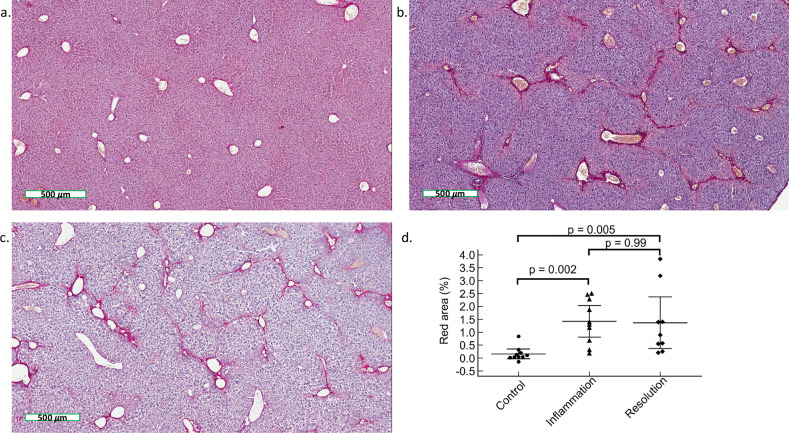
Picrosirius red-stained histological liver sections (scale bar: 500 µm) in a control mouse (**a**), and in mice during inflammation (**b**) and resolution (**c**) after CCl_4_ injection. Collagen deposition and bridging are observed in mice treated with CCl_4_ (**b**, **c**). Graph of hepatic collagen (% red area) quantification in control, inflammation, and resolution groups (**d**). Collagen percentage is significantly higher in mice treated with CCl_4_ than in control mice

On picrosirius red-stained histological sections, fibrosis was observed around centrilobular venules with septa formation in the livers treated with CCl_4_ (inflammation and resolution mouse groups). The percentage of liver fibrosis differed significantly between the three mouse groups (*p* = 0.001). Liver fibrosis percentage was significantly higher in the inflammation and resolution mouse groups than in the control group (1.4 ± 0.9% in the inflammation group and 1.4 ± 1.3% in the resolution group *versus* 0.2 ± 0.2% in the control group, *p* = 0.002 and *p* = 0.005, respectively). No significant difference in hepatic fibrosis percentage was observed between inflammation and resolution groups (*p* = 0.993) (Fig. 3). No significant correlation was observed between $\Delta {R}_{2}^{* }$ and fibrosis percentage (*p* = 0.278).

### Immunofluorescence

**Fig. 4:**
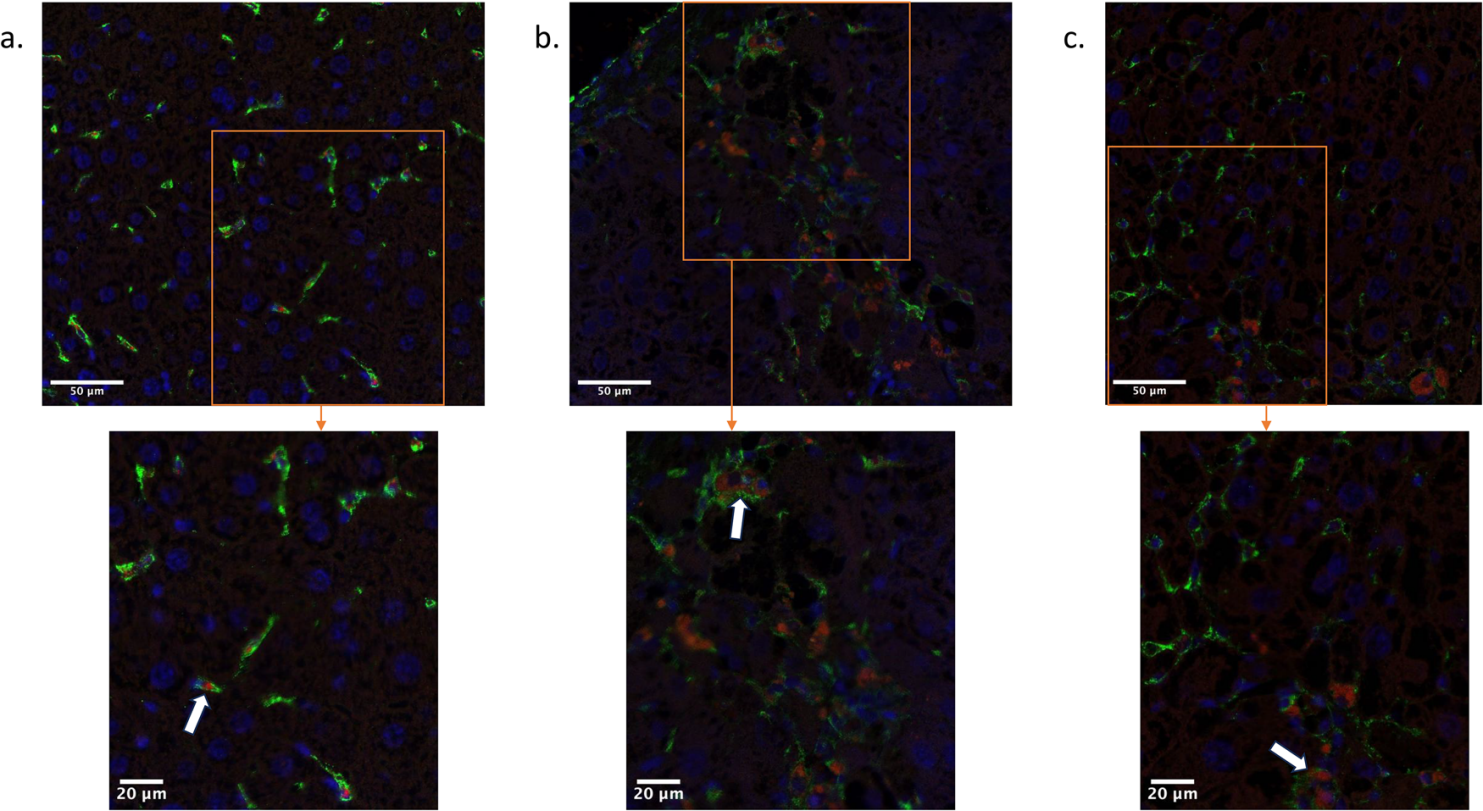
Liver images in a control mouse (**a**), in a mouse during inflammation (**b**), and in a mouse during resolution (**c**) obtained with confocal microscopy. Macrophages are colored in green, nuclei in blue, and fluorescent USPIOs appear in red. Arrows show internalization of fluorescent USPIO in macrophages. Increased USPIO uptake in macrophages is observed in the inflammation stage relative to the resolution stage and control. USPIO, Ultrasmall paramagnetic iron oxide

In the fibrotic mouse livers, fluorescent F4/80-positive cells accumulated around centrilobular venules and within fibrous septa (Fig. 4). This pattern was similar to the collagen distribution observed with picrosirius red staining. The USPIO rhodamine red fluorescence was discernible almost exclusively in colocalization with F4/80 green fluorescence. In the control group, F4/80-positive cells were homogeneously distributed in the liver lobules, and USPIO inside the macrophages was less discernible than in the inflammation and the resolution groups.

The USPIO fluorescence intensity, number of macrophages, and the mean USPIO fluorescence intensity per macrophage differed significantly between the three mouse groups (*p* < 0.0001, *p* = 0.002, and *p* < 0.0001, respectively).

**Fig. 5:**
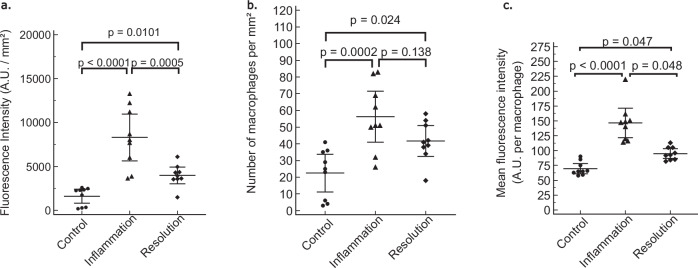
Quantification of USPIO fluorescence intensity (**a**), macrophage count (**b**), and fluorescence intensity (**c**) per macrophage of control, inflammation, and resolution groups. Fluorescence intensity, number of macrophages, and fluorescence intensity per macrophage are significantly higher in the inflammation group than in the control group. Fluorescence intensity and fluorescence intensity per macrophage are also significantly higher in the inflammation group than in the resolution group. USPIO, Ultrasmall paramagnetic iron oxide

The total USPIO fluorescence intensity (Fig. 5a) was significantly higher in the inflammation group (8.3 ± 3.5 × 10^3^ A.U./mm²) than in the control group (1.7 ± 1.0 ×10^3^ A.U./mm², *p* < 0.0001) and in the resolution group (4.2 ± 1.3 × 10^3^ A.U./mm², *p* = 0.0101). The total fluorescence intensity was also significantly higher in the resolution group than in the control group (*p* = 0.0005) (Fig. 5a).

The macrophage density (Fig. 5b) was significantly higher in the inflammation group (56 ± 20 macrophages/mm²) than in the control group (23 ± 14 macrophages/mm², *p* = 0.0002), and was not significantly different from that of the resolution group (42 ± 11 macrophages/mm², *p* = 0.138). Macrophage density was significantly higher in the resolution group than in the control group (*p* = 0.024).

The mean USPIO fluorescence per macrophage (Fig. 5c) was highest in the macrophages of the inflammation group (147 ± 32 A.U./macrophage), and differed significantly compared to the control group (71 ± 11 A.U./macrophage, *p* < 0.0001) and the resolution group (98 ± 14 A.U./macrophage, *p* = 0.048. Control and resolution groups were significantly different (*p* = 0.047).

**Fig. 6:**
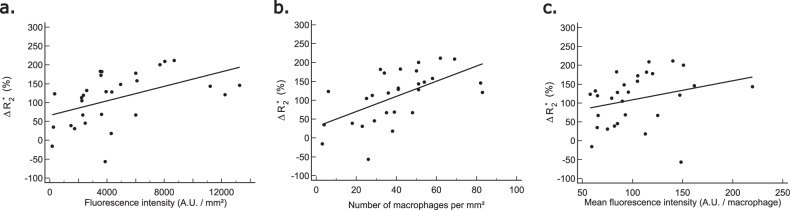
Correlations between $\triangle {R}_{2}^{* }$ (%) and USPIO fluorescence intensity (**a**: *r* = 0.58, *p* = 0.0011), number of macrophages per mm² (**b**: *r* = 0.67, *p* = 0.0001), and fluorescence intensity per macrophage (**c**: *r* = 0.40, *p* = 0.03)

$\Delta {R}_{2}^{* }$ measured with MRI was correlated with the USPIO fluorescence intensity (*r* = 0.58, *p* = 0.0011), the number of macrophages (*r* = 0.67, *p* = 0.0001), and the fluorescence intensity per macrophage (*r* = 0.40, *p* = 0.0311) (Fig. 6).

### Iron concentration

**Fig. 7:**
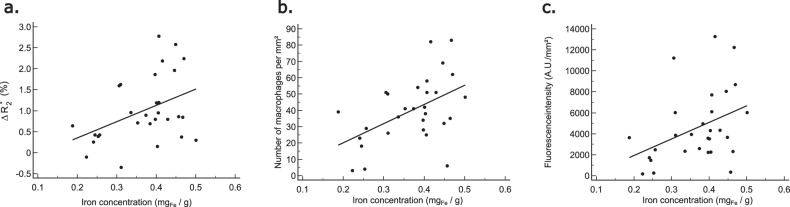
Correlations between inductively coupled mass spectrometry iron concentration and $\Delta {R}_{2}^{* }$ (**a**), number of macrophages per mm² (**b**), and total fluorescence intensity (**c**). Significant correlations are observed between iron concentration and $\Delta {R}_{2}^{* }$ (*r* = 0.39, *p* = 0.028), number of macrophages per mm² (*r* = 0.48, *p* = 0.013), and total fluorescence intensity (*r* = 0.46, *p* = 0.008)

Iron concentrations quantified with ICP-MS for the control, inflammation, and resolution groups were 0.34 ± 0.09 mg_Fe_/g, 0.39 ± 0.07 mg_Fe_/g, and 0.37 ± 0.09 mg_Fe_/g, respectively. The iron concentration tended thus to be higher in the inflammation group, intermediate in the resolution group, and lower in the control group, without significant differences (*p* = 0.271). Iron concentration was significantly correlated with $\Delta {R}_{2}^{* }$ (*r* = 0.39, *p* = 0.028), with the number of macrophages per mm² (*r* = 0.48, *p* = 0.013), and with total fluorescence intensity (*r* = 0.46, *p* = 0.008), but not fluorescence intensity per macrophage (*p* = 0.093, not shown) (Fig. 7).

## Discussion

We showed the feasibility of differentiating with USPIO-enhanced MRI the inflammation and resolution stages of hepatic injury in mice. With MRI, we observed statistically significantly higher liver $\Delta {R}_{2}^{* }$ in the inflammatory mice group than in the control group and in the resolution group. Similarly, with fluorescence imaging, we observed significantly higher total macrophage USPIO fluorescence in the inflammation group than in the two other groups. Moreover, with ICP-MS, there was a trend of higher iron concentration in the inflammatory group, although the differences with the two other groups were not statistically significant.

The lower differences observed between the control, inflammation, and resolution mice groups with ICP-MS relative to MRI may be explained by several reasons. First, ICP-MS is equally sensitive to exogenous iron (here, superparamagnetic USPIO) and endogenous iron (paramagnetic iron-containing proteins, including hemoglobin, transferrin, and ferritin). In contrast, MRI is more sensitive to the superparamagnetic USPIO through susceptibility effects. Moreover, these susceptibility effects are increased when USPIOs are clustered intracellularly, causing increases in $\Delta {R}_{2}^{* }$, not directly related to iron quantity. Finally, potential differences in the number of endogenous iron-containing proteins between the control, inflammation, and resolution groups might have partially obscured the differences between groups when measured with ICP-MS.

Globally, these results between groups and the significant correlations between $\Delta {R}_{2}^{* }$ and number of macrophages, mean and total fluorescence intensity, and iron concentrations, suggest that the transient increase in macrophage total activity during acute liver injury can be detected with USPIO-enhanced MRI. It should be noted that the mean and total USPIO fluorescence were significantly higher in the inflammatory group than in the control and resolution groups. Regarding the macrophage number, there was also a significant increase in the inflammatory group, but only a decreasing trend in the resolution group. This might be explained by the early time point of imaging (day 2) that we used to assess inflammation reversal [17].

Our MRI findings are in accordance with data from invasive methods in human samples and mouse models, showing that during liver injury, recruited monocyte-derived macrophages expand largely. These Ly6C^hi^ macrophages are proinflammatory and profibrogenic. In a second step, restorative Ly6C^low^ macrophages promote inflammation resolution and scar degradation [2, 4, 17]. It has been reported that the proinflammatory Ly6C^high^ macrophages have high expression of phagocytosis genes and display higher phagocytic capacity than the Ly6C^low^ pro-resolution macrophages [14, 17]. In mice with CCl_4_ induced liver injury, it has been shown that the proportion of proinflammatory, highly phagocytic Ly6C^hi^ macrophages strongly increased one day after cessation of injury and decreased thereafter, whereas the proportion of pro-resolution Ly6C^low^ macrophages was higher than that of proinflammatory macrophages between 2 and 4 days after treatment [14, 17]. These dynamics were similar to those we observed in our MRI and immunofluorescence study, namely higher hepatic macrophage number and uptake during the inflammation stage (day 1) and lower number and uptake during the resolution phase (day 2). In contrast, the hepatic collagen percentage at histological examination remained similar between the inflammation and resolution stages, as previously reported [2, 16].

Previous MRI studies with SPIO in chronic liver disease have reported decreased hepatic signal intensity variation and decreased $\Delta {R}_{2\,}^{* }$ after SPIO injection in metabolically-dysfunction-associated steatohepatitis (MASH) and cirrhosis [7–12]. This low signal intensity and low $\Delta {R}_{2}^{* \,}$ have been reported to be related to impaired macrophage uptake function of SPIO or decreased number of macrophages.

These previous static, one-time-point results differ from the dynamic, transient high $\Delta {R}_{2}^{* }$ that we observed during the inflammatory stage in liver injury. Moreover, several other reasons might explain the observed differences. First, most previous studies were performed with larger SPIOs than the USPIOs we used in the current study. It is known that various factors, including SPIO size, surface properties, and opsonization, influence nanoparticle pharmacokinetics and macrophage uptake [14, 22, 23]. Second, only early time points for post-contrast MRI (less than 30 min after SPIO injection) were used in most previous studies. During these early time points, intravascular and extracellular SPIOs (with different T_1_ and T_2_* relative to intracellular SPIOs) rather than intramacrophagic SPIOs may influence the MRI signal intensity [24]. Third, in steatotic liver disease, liver fat leads to errors in *R*_2_* estimation using an exponential signal model, unless fat-corrected *R*_2_* measurements are performed [25]. Interestingly, in steatotic liver disease, it has been reported that the signal intensity variation after SPIO injection was more associated with steatosis than inflammation [9, 11], showing the high influence of fat on signal intensity measurements and Δ*R*_2_* calculations [26, 27].

The bi-modal USPIOs we used in this study are a prototypical contrast agent designed for macrophage imaging. This compound is equivalent to P904 with the addition of rhodamine B for fluorescent detection. P904 has been previously studied in extrahepatic inflammatory diseases, showing high concentrations in the aortic wall and adipose tissue inflammation [18, 19].

Another approach for iron-enhanced MRI of liver inflammation and fibrosis is molecular imaging with targeted SPIO or iron that recognizes a specific protein, receptor, or biological process [28]. For example, a peptide-based USPIO targeted to αvß3 integrins expressed in activated hepatic stellate cells, has been developed to image fibrogenesis in CCl_4_-injured rats [29, 30]. More recently, MRI enhanced with Fe-PyC3A, an oxidatively activated probe, has been developed for liver inflammation imaging in mice [31]. Relative to these molecular approaches, untargeted USPIOs, as used in our small animal study, have already been used in clinical trials [12, 32, 33].

Our study has potential clinical implications for patients with diffuse liver diseases, including viral hepatitis and MASH. These diseases are classically diagnosed with histological analysis of liver biopsies. However, liver biopsy is infrequently performed nowadays because of its invasive nature, potential for sampling error, and lack of inter-rater reliability. Therefore, non-invasive alternatives to liver biopsy are needed to assess diffuse liver diseases. Ultrasound and MR elastography are established methods for assessing liver fibrosis severity in diffuse liver diseases [34, 35]. However, the diagnostic performance of elastography to assess liver inflammation remains debated [36–39]. The results of our animal study suggest that the inflammatory phase during liver injury may be detected with USPIO-enhanced MRI. If these results are duplicated in clinical trials, USPIO-enhanced MRI might be useful to diagnose MASH and inflammatory bouts in chronic viral hepatitis.

There are some limitations in our study. The liver sections analyzed with histopathology did not exactly match the analyzed MR images. However, both analyses were performed in the right lobe of the mice's livers.

Another drawback is that we did not subtype the hepatic macrophages, as this characterization was beyond the scope of this diagnostic radiology study. However, as explained above, the macrophage dynamics observed with MRI, immunofluorescence, and ICP-MS in our study were similar to those previously reported in molecular biology studies [2, 4, 17].

We have used a CCl_4_-induced toxic liver injury model, as it represents a robust and reproducible model of acute and chronic liver injury that has been previously used to assess liver macrophage dynamics [2, 17]. In future studies, the role of USPIO-enhanced MRI should be studied in other models of liver injury, including models of MASH.

In conclusion, our results in mice suggest that the inflammatory and resolution stages of hepatic injury can be assessed with USPIO-enhanced MRI. If confirmed in further studies, this suggests that USPIO-enhanced MRI might be useful for assessing hepatic inflammation dynamics.

## Data Availability

The datasets used and/or analyzed during the current study are available from the corresponding author on reasonable request.

## Abbreviations

CCl_4_: Carbon tetrachloride
ICP-MS: Inductively coupled mass spectrometry
MASH: Metabolically-dysfunction-associated steatohepatitis
PBS: Phosphate-buffered saline
RGB: Red, green, and blue
ROI: Region of interest
USPIO: Ultrasmall paramagnetic iron oxide
